# A chromosome-level genome assembly of *Coffea arabica* L. var. ‘Kona Typica’

**DOI:** 10.1038/s41597-025-05658-6

**Published:** 2025-07-29

**Authors:** Haomin Lyu, Jinjin Song, Yanbin Yin, Ming-Li Wang, Tracie Matsumoto, Vaishnavi Annabhemoju, Lyndel W. Meinhardt, Sunchung Park, Seunghyun Lim, Dapeng Zhang, Qingyi Yu

**Affiliations:** 1 Tropical Plant Genetic Resources and Disease Research Unit, Daniel K Inouye U.S. Pacific Basin Agricultural Research Center, Agricultural Research Service, U.S. Department of Agriculture, Hilo, Hawaii 96720 USA; 2 Hawaii Agriculture Research Center, Kunia, Hawaii 96759 USA; 3 Nebraska Food for Health Center, Department of Food Science and Technology, University of Nebraska-Lincoln, Lincoln, Nebraska 68588 USA; 4 Sustainable Perennial Crops Laboratory, Agriculture Research Service, U. S. Department of Agriculture, Beltsville, Maryland 20705 USA

**Keywords:** Genome duplication, Comparative genomics

## Abstract

*Coffea arabica* L. var. ‘Kona Typica’ is renowned for its premium cup quality, but its vulnerability to pests and diseases limits production. To accelerate cultivar improvement, we generated a chromosome-level genome assembly of ‘Kona Typica’ using PacBio HiFi sequencing and Hi-C scaffolding technology. The final assembly spans 1.13 Gb, with a scaffold N50 of 50.50 Mb, organized into 22 chromosomes. BUSCO assessment indicated a high completeness at 99.1%. We annotated 65,458 protein-coding genes and identified 1,073,545 interspersed repeats, accounting for 65.16% of the genome. Analysis of transposon insertion ages revealed that most long terminal repeat retrotransposons proliferated after the polyploidization event. This high-quality genome assembly of ‘Kona Typica’ provides a valuable resource for exploring coffee genomic evolution and genetic mechanisms of complex traits, facilitating genomics studies and the development of improved coffee cultivars with enhanced disease resistance and quality traits.

## Background & Summary

Coffee, belonging to the genus *Coffea* in the Rubiaceae family, is one of the most widely cultivated beverage crops in tropical and subtropical regions, renowned for its economic importance and cultural significance^1–3^. The genus *Coffea* originated in tropical and subtropical Africa and comprises approximately 131 species^4^. Among these, only two species, *Coffea arabica* (commonly known as Arabica) and *Coffea canephora* (commonly known as Robusta), are widely cultivated. Arabica coffee accounts for 60–70% of world production and is known for its smooth, well-balanced taste and lower caffeine content compared to Robusta coffee^5^.

Within the genus *Coffea*, *C. arabica* is the only tetraploid species (2n = 44) and is self-fertile, while all other *Coffea* species, including *C. canephora*, are diploid (2n = 22) and self-sterile. Despite its tetraploid nature, *C. arabica* exhibits diploid-like meiotic behavior. Studies suggest that *C. arabica* originated from a spontaneous hybridization event between the two diploid species, *C. canephora* and *C. eugenioides*, approximately 0.665 million years ago^6–9^.

*C. arabica* originated in southwestern Ethiopia, where its center of genetic diversity remains^10,11^. Cultivation of *C. arabica* began in Ethiopia approximately 1500 years ago. The first migration of *C. arabica* was from Ethiopia to Yemen as part of the prehistoric trade^11–15^. From Yemen, *C. arabica* spread to the Malabar coast of India and subsequently to Ceylon and Java in the late 17^th^ century^11–15^. A single *C. arabica* plant from Java was later transferred to the botanical garden in Amsterdam in 1706. Seedlings from this plant, subsequently named ‘Typica’, were further introduced to Martinique and from there to South America. Similarly, other *C. arabica* materials collected from Yemen were introduced to Reunion (formerly Bourbon Island) and from there reached South America as the cultivar ‘Bourbon’. These early introductions, involving a limited number of plants, significantly restricted the genetic diversity of Arabica coffee cultivars worldwide^16^.

Typica coffee was introduced to Hawaii from Guatemala in 1892, where it became the predominant variety in the Kona region^17^. Today, over 90% of coffee produced in Kona is known as ‘Kona Typica’. ‘Kona Typica’ is renowned for its exceptional quality and flavor, largely attributed to the unique geographic and climatic conditions of the region. However, despite its superior taste and quality, ‘Kona Typica’ is highly susceptible to pests and diseases, such as coffee leaf rust (CLR) and coffee berry disease (CBD). While Hawaii was among the last coffee-producing regions free of CLR, the disease was detected on Maui in February 2020 and subsequently spread to all other Hawaiian coffee-growing islands^18^. Breeding programs for Typica coffee are prioritizing the improvement of disease and pest resistance to mitigate yield losses and economic impact. However, the lack of comprehensive molecular data on resistance genes remains a significant barrier to progress in coffee breeding programs.

The first draft genome of coffee was published in 2014, sequenced from a doubled haploid Robusta coffee accession using a combination of Sanger, 454, and Illumina technologies^6^. With advancements in sequencing technology, several coffee genomes have since been assembled. Salojärvi *et al*. generated chromosome-level assembles for the di-haploid Arabica line ET-39, the previously sequenced doubled haploid Robusta accession, and the wild *Eugenioides* accession Bu-A^7^. Additionally, Scalabrin *et al*. constructed a reference genome for ‘Bourbon’ using Oxford Nanopore Technologies (ONT) long reads and Hi-C scaffolding^8^. Similarly, a chromosome-level assembly for ‘Geisha’ was developed by integrating ONT and PacBio long-read sequencing^19^.

‘Typica’ is one of the oldest and most culturally and genetically significant lineages of Arabica coffee. Deciphering the genome structure and organization of ‘Kona Typica’ coffee is critical for unraveling the genetic networks regulating important traits, thereby facilitating the development of superior varieties. In this study, we present a chromosome-level genome assembly of ‘Kona Typica’ coffee constructed using PacBio long-read sequencing and Omni-C data. The resulting genome comprises 22 chromosomes with a total size of 1.13 Gbp and a scaffold N50 of 50.50 Mbp. This high-quality reference genome provides valuable insights into the genomic features of ‘Kona Typica’ coffee and contributes to understanding the evolutionary history and domestication process of Arabica coffee genome.

## Methods

### Plant materials

The Arabica coffee variety ‘Kona Typica’ KO34 was planted in the field at Hawaii Agriculture Research Center, Kunia, Oahu. Young leaf tissue of KO34 was harvested for high molecular weight DNA extraction.

### Library preparation for PacBio Sequencing and Omni-C sequencing

High molecular weight genomic DNA was extracted from isolated nuclei using lysis buffer with proteinase K, followed by phenol–chloroform extraction and isopropanol precipitation to purify DNA with minimal shearing and degradation. PacBio HiFi sequencing library was prepared using the SMRTbell Express Template Prep Kit 2.0. Major steps include end-repair, adapter ligation, and nuclease treatment. The DNA library was sequenced on a PacBio Sequel II System. A total of 11,084,430 HiFi reads were generated, with an average length of 12,224 base pair (bp), a maximum length of 49,767 bp, and a total length of 135,499,623,700 bp (Table 1). For Hi-C sequencing, the library was prepared using the Omni-C™ Technology and sequenced on an Illumina HiSeqX system at Dovetail Genomics. Detailed method follow our previous publication^20^. A total of 243,981,225 pairs of Omni-C reads (2 × 150 bp) were obtained, with a total length of 73,194,367,500 for genome assembly (Table 2).

**Table 1:** General Statistics of PacBio HiFi sequencing data used in ‘Kona Typica’ genome assembly.

Metrics	Value
Number of reads	11,084,430
Total bases (bp)	135,499,623,700
Minimum length of reads (bp)	101
Maximum length of reads (bp)	49,767
Average length of reads (bp)	12,224

**Table 2:** Summary statistics of Omni-C sequencing data used in ‘Kona Typica’ genome assembly.

Metrics	Value
Number of read pairs	243,981,225
Average length of reads (bp)	150
Total bases (bp)	73,194,367,500
GC (%)	42

### Genome assembly and quality assessment

**Fig. 1:**
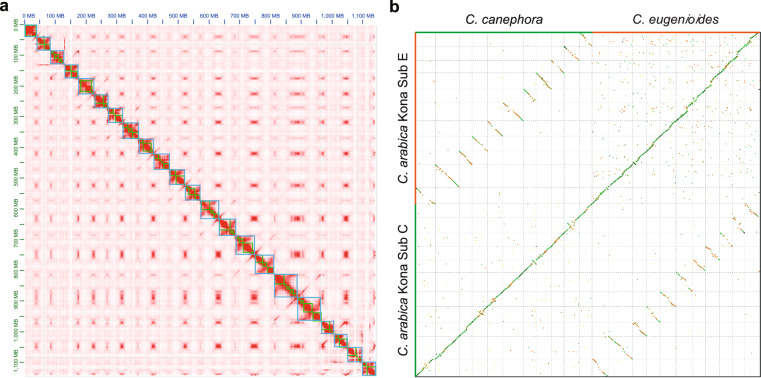
Chromosome-level scaffolding and chromosome assignment. (**a**) Genome-wide Hi-C interactive heatmap showing 22 pseudo chromosomes. (**b**) Genome alignment between the two subgenomes of ‘Kona Typica’ (Sub C and Sub E) and their progenitor species *C. canephora* and *C. eugenioides*.

The ‘Kona Typica’ genome was assembled using Hifiasm (v0.15.4-r347)^21^ with the parameter ‘-l 0’. Omni-C reads were used to phase haplotypes. The resulting draft genome is about 1.21 Gb, consisting of 2,138 contigs with an N50 of 49.07 Mb (Table 3). Omni-C reads were aligned to the contig-level genome assembly using chromap (v0.2.5-r473)^22^. YaHs (v1.2a.1)^23^ were used for scaffolding with default parameters. Hi-C contact matrices and editable Hi-C maps were generated using Juicer (v1.1)^24^, and Juicerbox (v1.11.08)^24^ facilitated visualization and manual error correction of Hi-C maps throughout the genome assembly. Minimap2 (v2.26-r1175)^25^ and GS-GapCloser (v1.2.1)^26^ were used to fill the N-gap. Purge_Haplotigs (v1.1.2)^27^ was used to eliminate haplotig duplications following the pipeline protocol. The final assembly consisted of 967 scaffolds with a total length of 1,172,725,641 bp and an N50 of 50.50 Mb. These scaffolds were anchored to 22 chromosomes with an accumulative length of 1,135,979,557 bp (Table 3, Fig. 1a). BUSCO (v.5.2.2)^28^ analysis was conducted to assess the quality of the assembled genome and revealed high genome completeness, with 99.1% of BUSCO genes identified: 80 (5%) complete single copy genes, 1,519 (94.1%) complete duplicated genes, 6 (0.4%) fragmented genes, and 9 (0.5%) missing genes (Table 3). The 22 chromosomes were grouped into subgenome C (subC) and subgenome E (subE) (Fig. 1b), corresponding to *C. canephora* and *C. eugenioides* origins, respectively. A summary of the chromosome-level assembly is shown in Table 4.

**Table 3:** Summary statistics of the ‘Kona Typica’ genome assembly.

Assembly characteristics	Value
Number of contigs	2,138
Contig size (bp)	1,214,654,331
Contig N50 (bp)	49,070,996
Number of scaffolds	967
Scaffold size (bp)	1,172,725,641
Scaffold N50 (bp)	50,495,782
Scaffold N90 (bp)	40,978,299
Number of chromosomes	22
Total length of anchored sequences on chromosomes (bp)	1,135,979,557
Total length of unanchored sequences (bp)	36,746,084
Proportion of unanchored sequences	3.13%
Complete BUSCOs (Number / %)	1,599 / 99.1
Complete and single-copy BUSCOs (Number / %)	80 / 5.0
Complete and duplicated BUSCOs (Number / %)	1,519 / 94.1
Fragmented BUSCOs (Number / %)	6 / 0.4
Missing BUSCOs (Number / %)	9 / 0.5

**Table 4:** Summary of the chromosome-level genome assembly of ‘Kona Typica’.

Subgenome C		Subgenome E	
Chromosome number	Length (bp)	Chromosome number	Length (bp)
Chr01C	58,472,035	Chr01E	54,600,467
Chr02C	75,892,403	Chr02E	74,242,642
Chr03C	44,604,901	Chr03E	44,964,447
Chr04C	51,937,631	Chr04E	48,882,825
Chr05C	49,066,673	Chr05E	50,495,782
Chr06C	64,302,159	Chr06E	61,255,350
Chr07C	40,048,965	Chr07E	43,493,574
Chr08C	44,908,166	Chr08E	52,505,707
Chr09C	44,609,420	Chr09E	40,978,299
Chr10C	52,464,068	Chr10E	47,015,880
Chr11C	43,937,275	Chr11E	47,300,888

### Identification and annotation of repetitive sequences

**Fig. 2:**
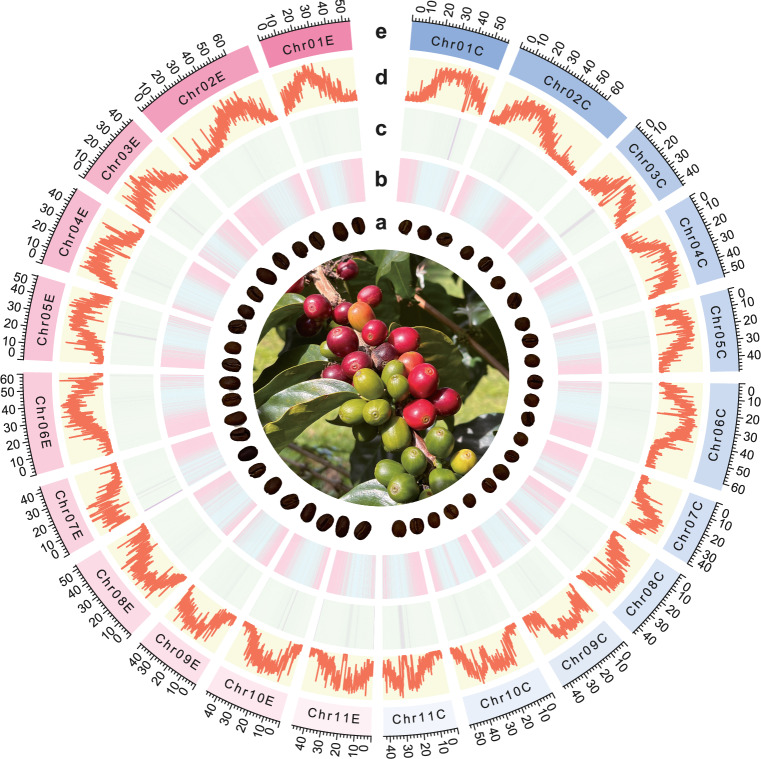
Circos plot illustrating features of the chromosome-level assembly of *Coffea arabica* L. var. ‘Kona Typica’. (**a**) Comparison of roasted Arabica (left) and Robusta (right) coffee beans. Arabica beans are typically oval-shaped, flat, and have a curved crease, while Robusta beans are generally rounder, smaller, and have s straight crease. (**b**) gene density (low to high, indicated by blue to red gradient); (**c**) GC content; (**d**) TE content; (**e**) chromosomes, with megabase pair (Mbp) scale shown above. All density plots were generated using a 100 kbp sliding window size.

Transposable elements (TEs) in the ‘Kona Typica’ genome were identified and annotated using RepeatMasker (v4.1.0)^29^ with *de novo* libraries constructed using RepeatModeler (v1.0.8)^30^ and EDTA (v2.2.0)^31^. A total of 1,073,545 interspersed repeats, accounting for 65.16% of the ‘Kona Typica’ genome, were annotated, including long terminal repeat (LTR) and non-LTR retrotransposons, and terminal inverted repeat (TIR) and non-TIR DNA transposons (Table 5, Fig. 2). Detailed information about the number, total length, and percentage for individual classes of TEs is listed in Table 5. LTR-Gypsy retrotransposons were the most abundant, accounting for 26.41% of the genome (Table 5).

**Table 5:** Overview of repetitive sequence composition in the ‘Kona Typica’ genome.

Type			Count	Length (bp)	% in genome
Class I: Retrotransposons	LTR	Copia	67,410	64,426,902	5.49
		Gypsy	251,172	309,760,938	26.41
		unknown	281,031	209,339,703	17.85
	Non LTR	LINE_element	3,312	1,526,179	0.13
		unknown	461	162,023	0.01
	Total retrotransposons		535,976	585,215,745	49.89
Class II: DNA transposons	TIR	CACTA	42,685	15,075,054	1.29
		Mutator	123,251	42,355,413	3.61
		PIF_Harbinger	15,271	4,847,098	0.41
		Tc1_Mariner	16,452	4,553,741	0.39
		hAT	37,652	15,790,575	1.35
	Non TIR	helitron	87,867	31,426,976	2.68
	Total DNA transposons		323,178	114,048,857	10.00
repeat_region			146,981	64,921,190	5.54
Total interspersed repeat			1,073,545	764,185,792	65.16

**Fig. 3:**
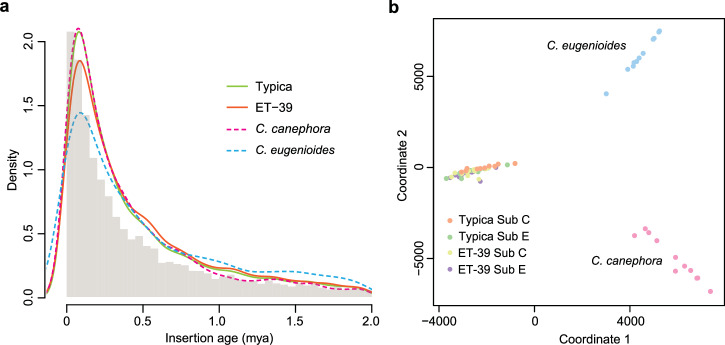
Comparative analysis of transposon insertion ages among four coffee genomes. (**a**) Density distribution of LTR-RT insertion ages in a scale of million years ago (mya). The bar plot presents LTR-RT insertion ages in the ‘Kona Typica’ genome. (**b**) Chromosome clustering of ‘Kona Typica’ (Sub C and Sub E), *C. canephpra*, and *C. eugenioides* genomes using Similarity Matrix analysis.

Analysis of transposon insertion ages in *C. eugenioides*, *C. canephora*, *C. arabica* var. ‘ET-39’, and *C. arabica* var. ‘Kona Typica’ genomes showed variations in transposon accumulation and removal rates among the four genomes, but all four genomes exhibited a sharp increase in LTR proliferation around 0.05–0.15 million years ago (mya) (Fig. 3a). This finding was supported by Similarity Matrix Analysis of LTR elements^32,33^, which showed that the two subgenomes of ‘Kona Typica’ clustered separately from its diploid progenitors, *C. eugenioides* and *C. canephora* (Fig. 3b), suggesting most LTRs in ‘Kona Typica’ genome proliferated post- polyploidization (~0.665 mya).

### Prediction of protein-coding genes

Protein-coding genes were predicted using a combination of homology-based, RNA-seq-guided and *ab initio* gene prediction approaches. For homology-based gene prediction, protein sequences from published genomes of *C. arabica*^8^, *C. eugenioides*^8^, *C. canephora*^6^, *C. humblotiana*^34^, and *Leptodermis oblonga*^35^ were used. RepeatMasker^29^ was first applied to mask TEs, followed by homology-based gene prediction using GeMoMa (v1.9)^36^ with default parameters. For RNA-seq guided gene prediction, RNA-seq data were retrieved from NCBI database and mapped to the reference genome to identify coding genes. Hisat2 (v2.2.0)^37^ and TACO (v0.7.3)^38^ were used to align short RNA-seq reads to the reference genome and reconstruct full-length transcripts. Putative coding regions were identified using TransDecoder (v5.7.0) (https://github.com/TransDecoder/TransDecoder). *Ab initio* gene prediction was performed using Augustus (v3.3.2)^39^, GeneMark-ET (v4.59)^40^ and Braker2 (v2.1.2)^41^ with the mapping files of the RNA-seq data. Finally, EVidenceModeler (v2.0.0)^42^ was used to integrate predictions from all three methods. A total of 65,458 protein-coding genes were identified in the assembled genome (Table 6).

**Table 6:** Summary of genome-wide annotation of functional genes.

Metrics	Number
Protein coding gene	65,458
eggNOG-mapper	52,765
PANTHER	56,658
Pfam	49,905
InterPro	51,401
GO (eggNOG-mapper)	24,784
KEGG (eggNOG-mapper)	26,173
KEGG (Automatic Annotation Server)	17,045

### Functional annotation of protein-coding genes

Functional annotation of protein-coding genes was achieved through a multi-step approach. Initially, eggNOG-mapper (v5.0)^43^ was used to assign functional annotations based on the eggNOG database. Following this, InterProScan (v5.44-79.0)^44^ was conducted to predict protein domains and assign Gene Ontology (GO) terms. Finally, KEGG Automatic Annotation Server^45^ was used for Kyoto Encyclopedia of Genes and Genomes (KEGG) annotation to reveal the metabolic and signaling pathways represented by the genes. This process resulted in the annotation of 52,765, 56,658, 49,905, and 51,401 genes using eggNOG-mapper, PANTHER, Pfam and InterPro database, respectively (Table 6). Furthermore, eggNOG-mapper identified 24,784 genes with GO terms and 26,173 genes with KEGG Orthology KO) terms (Table 6).

### Identification of genetic variations

To identify genetic variations within the ‘Kona Typica’ genome, PacBio HiFi reads were aligned to the assembled ‘Kona Typica’ genome using pbmm2 (https://github.com/PacificBiosciences/pbmm2) and the output was used for the identification of structural variations using pbsv (https://github.com/PacificBiosciences/pbsv). This analysis detected 6,873 single nucleotide polymorphisms (SNPs) and 240,883 insertion-deletions (INDELs) in the genome (Table 7).

**Table 7:** Summary of genetic variations within the ‘Kona Typica’ genome (Sub C and Sub E).

Type	Number
SNP	6,873
INDEL	240,883
Total	247,756

## Data Records

PacBio HiFi and Hi-C sequencing reads have been deposited in the National Center for Biotechnology Information (NCBI) Sequence Read Archive (SRA) under project PRJNA1215012 and SRA accession number SRP560045^46^. The chromosome-level genome assembly is available in NCBI GenBank under accession number GCA_049114775.1^47^. The genome assembly, annotation, and genetic variation files for ‘Kona Typica’ genome have been deposited in figshare^48^. The genetic variation data has also been deposited in the Genome Variation Map (GVM) in National Genomics Data Center under accession number GVM001114^49^.

## Technical Validation

Genome assembly completeness was assessed using BUSCO (v5.2.2) against the embryophyta_odb10 orthologous database, which comprises 1,614 conserved single-copy genes^50^. This analysis revealed a 99.1% completeness score for the ‘Kona Typica’ genome. Additionally, Merqury (v1.3) program^51^ was used to assess genome assembly with HiFi reads, yielding a high consensus quality value (QV) of 57.72. These results demonstrate that the high quality of the *C. arabica* var. ‘Kona Typica’ genome assembly quality, characterized by exceptional completeness.

## Data Availability

All software and pipelines used in this study were executed according to the developers’ instructions, as referenced. Custom parameters are detailed in the main text.
